# Cost analysis of female breast cancer in Antigua and Barbuda: a prevalence based study

**DOI:** 10.3389/fpubh.2026.1779404

**Published:** 2026-03-17

**Authors:** Andre A. N. Bovell, Cebisile Ngcamphalala, Roxanne Brizan-St. Martin, Jabulani Ncayiyana, Themba G. Ginindza

**Affiliations:** 1 Discipline of Public Health Medicine, School of Nursing and Public Health, University of KwaZulu-Natal, Durban, South Africa; 2 Health and Social Transformation Unit, Organisation of Eastern Caribbean States, Castries, Saint Lucia; 3 Cancer & Infectious Diseases Epidemiology Research Unit (CIDERU), College of Health Sciences, University of KwaZulu-Natal, Durban, South Africa

**Keywords:** Antigua and Barbuda, breast cancer, costing analysis, costs, economic, healthcare costs, prevalence-based

## Abstract

**Background:**

Female breast cancer still represents a substantial public health obstacle in many countries. The condition remains costly for healthcare providers and imposes heavy burdens on healthcare systems in countries globally. There is a significant gap in information regarding the economic burden of carcinoma of the breast affecting the women of Antigua and Barbuda. Consequently, this research aims to quantify the costs related to female breast carcinoma from the perspective of the healthcare provider in the country.

**Methods:**

This study employed a prevalence-based cost of illness methodology. Data on female breast cancer were collected from four research sites in Antigua and Barbuda for the years 2017 to 2021 to calculate the average yearly prevalence. Both top down and bottom up costing methods were employed to compute direct medical costs, using the price levels from 2021 and converting the amounts to United States Dollars.

**Results:**

Estimated total annual direct medical costs for female breast carcinoma was USD3.1 million. Treatment for clinical stages I to IV accounted for 78% of costs. Our leading contributors to annual direct medical costs were treatment (USD2,458,305.82), post-treatment care (USD390,474.79), and diagnosis and imaging (USD143,045.38). Overall direct medical unit costs was estimated at USD177,618.02, with lead drivers being surgery, systemic therapy, and ‘other complications of treatment’.

**Conclusion:**

Our study presented findings regarding direct medical costs of female breast carcinoma in Antigua and Barbuda. Our cost estimates appeared considerable given the local context. These findings provided a reference for informing health policy, advising on resources allocation, and encouraging cost containment in female breast cancer management in Antigua and Barbuda.

## 1 Introduction

Breast cancer is the most commonly diagnosed invasive cancer in women worldwide (1). Globally, female breast cancer is among the leading cause of cancer incidence and deaths, being responsible for 11.6% of all diagnosed cancer cases, and 6.9% of all cancer deaths in 2022 alone (1). The incidence rates for this health disorder have markedly exceeded other carcinomas in high income and low-middle income countries to include several in Africa, Asia, and South America (1). This cancer ranks first in incidence and mortality in several countries of the Caribbean, such as in Antigua and Barbuda (1, 2).

Given its high prevalence and generally long-term survivorship needs, this malignant tumor is classified as one of the most expensive cancers globally (3–5), accounting for an approximately 7.7% of an Intl$ 25.2 trillion global economic cost for cancers (5).

Across countries of the world, the direct medical costs of diagnosing, detecting and managing female breast carcinoma varies widely, with values as high as USD 16.5 billion in the United States in 2010 (6), to lows of USD 339.22 million in South Korea (7), and between USD 420 and 470 million in Poland for the period 2017–2019 (8). In Latin America and the Caribbean, the pooled weighted average direct medical costs per patient ranged from a low of Intl$13,179 for stage I disease to a high of Intl$28,910 for stage IV disease (9), thus highlighting the tremendous cost of breast cancer care in a region consisting of countries with both developing and emerging economies (10).

Several studies have utilized the prevalence-based cost of illness approach to estimate the economic burden of carcinoma of the breast. These include studies by Matsumoto and colleagues to evaluate the costs of breast cancer in Japan (11), Jain and Mukherjee to examine breast cancer burden in households in Punjab India (12), and Afkar et al. to assess the cost of breast cancer in Iran (13). Unlike in countries of North America, Europe, Latin America, and elsewhere, where there exists an abundance of published evidence on the direct medical costs of breast cancer, which policymakers could find useful in resource allocation and budgetary estimation practices alongside measures to enhance availability of therapeutically effective treatment for carcinoma of the breast, such studies are absent in the English-speaking Caribbean (4, 9, 14).

Despite its status as an emerging and developing economy (15), and aside from a predicted increase in cases of female breast cancer in the country for years to come (16), based on our review of the literature, no previously published work has addressed the economic burden of carcinoma of the breast in Antigua and Barbuda. To address our observed gap, this research was undertaken to quantify the economic burden of female breast carcinoma on the island by calculating and presenting an analysis of its direct medical cost using the prevalence-based approach.

## 2 Materials and methods

### 2.1 Research design, setting, and population

We conducted a retrospective, prevalence-based cost of illness analysis from the healthcare provider’s perspective, that utilized some data and findings stated in published sources, namely ‘Incidence, trends and patterns of female breast, cervical, colorectal and prostate cancers in Antigua and Barbuda, 2017-2021: a retrospective study’ (16), ‘The economic burden of prostate cancer in Antigua and Barbuda: A prevalence-based cost of illness analysis from the healthcare provider perspective’ (17), and ‘Cost analysis related to diagnosis, treatment and management of cervical cancer in Antigua and Barbuda: A prevalence-based cost of illness study’ (18), to quantify the direct medical costs for female breast cancer in Antigua and Barbuda, an English-speaking Caribbean island, where 53% of the population are women and where public healthcare is financed through statutory deductions and government subventions (19, 20).

As stated elsewhere, the study population comprised women, older than 18 years, and diagnosed with an initial tumor of the breast between 01 January 2017, to 31 December 2021 (16). Further, data on each patient, heretofore classified according to the International Classification of Diseases, Tenth Revision (C50) (21), was abstracted from medical records of the Sir Lester Bird Medical Centre (SLBMC), Antigua and Barbuda, The Cancer Centre Eastern Caribbean (TCCEC), and the Medical Benefits Scheme (MBS) (16). Additional information regarding female breast cancer-related mortality came from the Health Information Division in the Ministry of Health, Wellness and the Environment, Antigua and Barbuda (HID) (16). The four study sites were selected based on their relevance, as they all contribute significantly to recorded cancer diagnoses in Antigua and Barbuda (16). Patients with recurrent female breast cancer were not included in this study (16). This approach was undertaken to ensure that the study provides accurate estimates of direct medical costs related to initial breast cancer diagnosis and treatment, thereby enabling consistency in the analysis and providing a clear understanding of the economic burden of female breast cancer care in the country (16, 22).

### 2.2 Summary of female breast cancer management in Antigua and Barbuda

Cancer care in Antigua and Barbuda is generally influenced by the World Health Organization (WHO) suggested strategies on cancer control and prevention (23). With this framework, and utilizing adapted National Comprehensive Cancer Network Clinical Practice Guidelines (NCCN Guidelines), which provide suggestions for optimizing care and enhancing treatment outcomes, several cancers are managed on the island (24).

**Figure 1:**
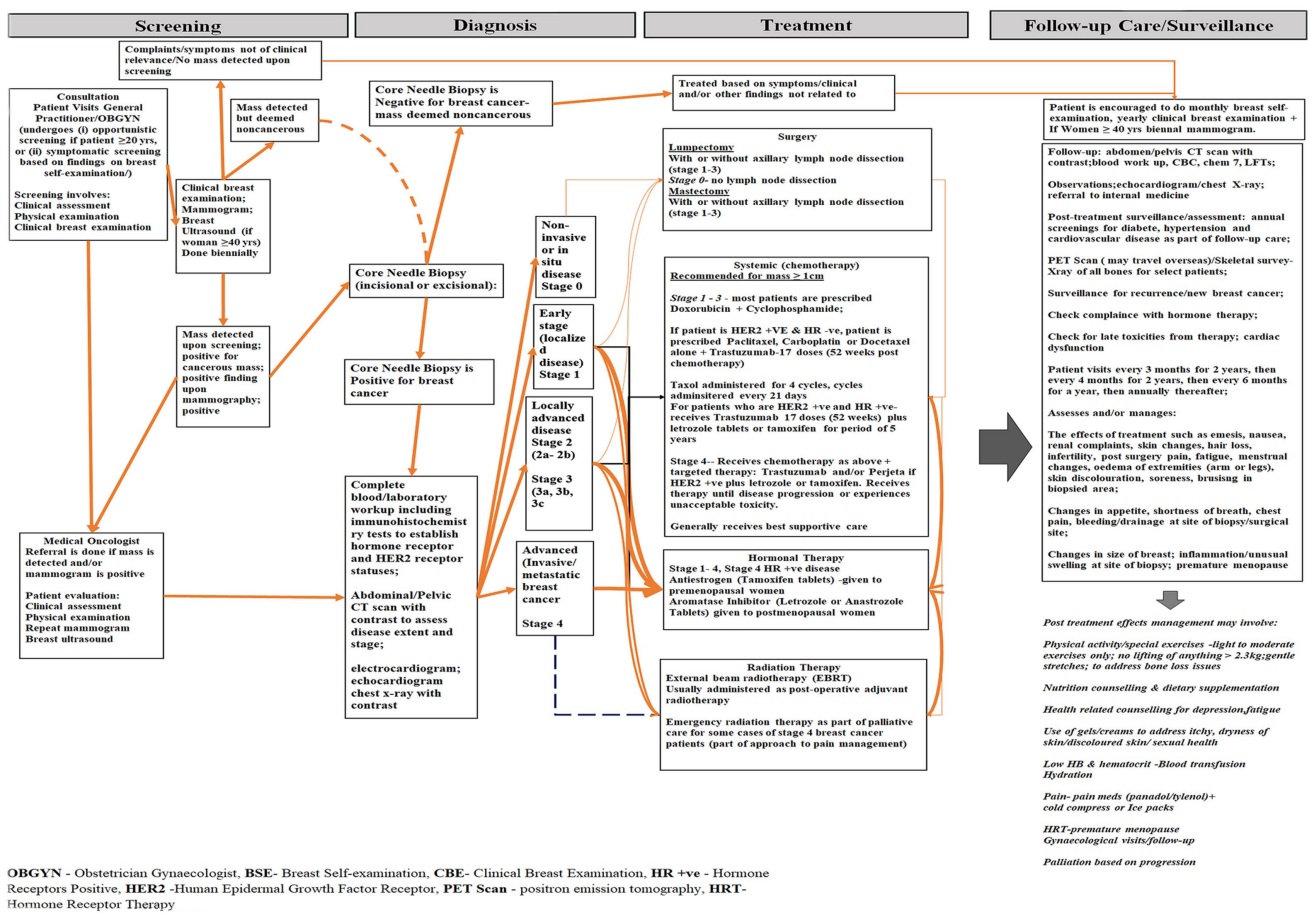
Illustrative diagram showing the main components of care related to the management of female breast cancer in Antigua and Barbuda (2017–2021).

Since patient care is needs-based and may devolve into inpatient and outpatient care, female breast cancer management usually starts with patients visiting a general practitioner or gynecologist, followed by subsequent referral to a medical oncologist based on clinical findings ascertained via asymptomatic or symptomatic screening (Figure 1). Screening, although it may stem from a patient conducting a breast self-examination, relies primarily on a clinical breast examination complemented using radiological techniques (imaging studies), namely mammography and/or breast ultrasonography, which forms the basis of a diagnosis of breast cancer. Should the findings from the imaging studies be positive, then a clinical diagnosis requiring either a core needle or Tru-cut biopsy is performed (25). If the biopsy returns positive, the patient is referred to a medical oncologist, where the disease is then staged. That is, in addition to the results of the biopsy, this involves the use of plain X-rays, computer tomography scan with contrast, scan of the abdominal/pelvic region, and immunohistochemistry tests (25). Collectively, this determines whether the disease is non-invasive (*in situ*), localized (early stage), localized to locally advanced, locally advanced, or advanced (metastatic). Breast cancer treatment in women is influenced by (a) both stage and extent of the malignancy (size, location and pathological characteristics), (ii) the age of the affected patient, and (iii) the person’s general health (26). Hence, it may comprise any of and/or a combination of (a) surgery (lumpectomy, mastectomy: with or without axillary lymph node dissection) (b) systemic therapy (chemotherapy), (c) radiotherapy (external beam radiotherapy), and (d) hormonal therapy (25). Additionally, some patients may require magnetic resonance imaging and positron emission tomography (PET scan), the latter of which is absent from the healthcare system in the nation (18, 27). Patients requiring PET scan services may receive treatment in health facilities abroad (20). Access to overseas care for female breast cancer patients are supported by the Medical Benefits Scheme (20).

Further, and in terms of oncological care, experts stated that breast cancer cases among women in Antigua and Barbuda were staged in accordance with the *American Joint Committee on Cancer (AJCC) 8th Edition Staging Manual* (16, 17).

Concerning this study, cost estimations across all components of care in female breast cancer management were obtained from 2021 market prices, reflecting charges from private clinics, diagnostic laboratories, and SLBMC unsubsidized pricing (Table 1) (17, 18, 26). Our price level was chosen to allow for there to be consistency and standardized comparability of the cost data over the study period, while accounting for possible distortions from inflation and other economic factors (22).

**Table 1:** Data variables and source of costs regarding screening, management and treatment of female breast cancer.

Identified Parameters	Source of Data	Source of Price
An estimate of women diagnosed with breast carcinoma	Aggregate count of patients obtained from records of Eastern Caribbean Cancer Centre, the Sir Lester Bird Medical Centre and Medical Benefits Scheme for the period 2017–2021.	N/A
Screening		
Consultation	Expert input and data was obtained from Medical Benefits Scheme, focusing on reimbursement claims, Board approved funding, as well as information from Billing and Finance of the Sir Lester Bird Medical and private laboratories across Antigua and Barbuda.	Prevailing price
Clinical assessment		Prevailing price
Physical examination		Prevailing price
Diagnostic evaluation and imaging		
Mammography	Expert input and data was obtained from Medical Benefits Scheme, focusing on reimbursement claims, Board approved funding, as well as information from Billing and Finance of the Sir Lester Bird Medical and private laboratories across Antigua and Barbuda.	Prevailing price
Breast ultrasonography		Prevailing price
Medical laboratory - Complete Blood Panel		Prevailing price
Histopathology		Prevailing price
Diagnostic imaging (CT, X-ray, ECG/EKG)		
Treatment		
Surgery	Expert inputs from Consultant Gynaecologist, Medical Oncologist, Senior Oncology Nurses at Eastern Caribbean Cancer Centre and the Sir Lester Bird Medical Centre, pharmacists, along with inputs and data from Medical Benefits Scheme, MBS-Board approved funding, as well as information from Billing and Finance of the Sir Lester Bird Medical Centre, private surgery, and manager of Operating Room at Sir Lester Bird Medical Centre.	Prevailing price
External Beam Radiation Therapy		Prevailing price
Systemic therapy (Chemotherapy)		MBS Board-Approved Funding/Prevailing price
Hormonal Therapy		Prevailing price
Post-treatment adverse events relieving health services		
	Expert inputs from Consultant Medical Oncologist/Gynaecologist, along with other inputs and data was obtained from Eastern Caribbean Cancer Centre, Medical Benefits Scheme, focusing on reimbursement claims, Board approved funding, as well as information from Billing and Finance of the Sir Lester Bird Medical Centre and private surgery.	Prevailing price
Thrombosis prevention		Prevailing price
Renal Impairment		Prevailing price
Reduced Haemoglobin levels		Prevailing price
Infection Control		Prevailing price
Additional treatment-related issues		Prevailing price
Supplementary direct costs		
Clinical Nutrition Consultation	Expert inputs from pharmacy, nutrition, oncology and gynaecology. Along with inputs and data from the Eastern Caribbean Cancer Centre, Medical Benefits Scheme reimbursement claims, and Board approved funding, as well as information from Billing and Finance of the Sir Lester Bird Medical Centre.	Private: Prevailing price
Mental Health Consultation		Prevailing price
Pharmaceutical Services		Prevailing price
Diagnostic Imaging (PET scan performed abroad)		MBS Board-Approved Funding/Prevailing price
Medical Response Package		
Travel Support and Housing for Overseas Medical Care		MBS Board-Approved Funding/Prevailing price
Local Transport Services		Prevailing price
Overhead Costs		Prevailing price

### 2.3 Calculating direct medical costs

This prevalence-based cost of illness study provided an estimate of the direct medical cost of female breast cancer conducted from the healthcare provider’s perspective (28). Direct medical costs was considered for its advantageousness in assessing economic burden in a healthcare environment (29, 30). Medical care interventions required in female breast cancer management (screening, diagnosis, treatment and management) were identified (micro-costing approach), quantified and valued per patient and extrapolated using prevalence data to estimate national costs (26, 31). Data were collected retrospectively using patient charts and an excel form (17, 26).

Direct medical costs were classified as recurrent costs (31, 32), encompassing personnel, transportation, consumable supplies (both medical and non-medical), treatment, administrative services, and overheads (31, 33). Using an approach discussed by Ginindza et al. (31), the annual number of female breast cancer patients requiring care in Antigua and Barbuda was estimated by subtracting deaths from the total patient population and dividing our answer by 5 (31).

Direct medical costs were then derived by multiplying the estimated patient number by the per-patient resource costs (17). The overall costs denoted a sum of all care-related parameters (17).


Direct Medical Costs of Female Breast Cancer(dc)=∑(Ci×Ri)

*Ci* is number of patients in need of health care.

*Ri* is the required health care resources unit costs per patient.

*dc* is the total costs.

For comparative cost analysis and to directly quantify the effect of unrecorded patients within our study sites, we utilized an approach mentioned in previous studies (18, 31). We adjusted upwards the annual patient population by 25%, and recalculated the total costs (31). Direct medical unit costs were also raised by 50%, with total annual costs re-estimated (18). We also reduced the treatment costs in our original model by 50% while holding the other parameters constant (18). We examined and reported the resulting changes in total annual costs (18).

### 2.4 Acquisition and presentation of cost data

Costs data pertaining to breast cancer screening, diagnostic imaging, treatment and follow-up care were systematically collected using an electronic dataset structured in accordance with the layout of routine records of the study sites (18, 34). Our tool was developed on the basis of the country’s breast cancer care pathway, encompassing screening, diagnosis, imaging, and treatment (Figure 1) (35).

Information on direct medical costs was obtained from reimbursement claims archived at the Medical Benefits Scheme, and covering records for laboratory, imaging, pathology, ultrasound, surgical, medication, hospitalization, and related services rendered to beneficiaries by private health institutions (17, 20). Supplemental information was received from private laboratories and clinics (17).

Reported costs were expressed in 2021 US dollars. Conversion was done using the national consumer price index (CPI) for 2021 and the prevailing exchange rate (1 USD = 2.7169 XCD). The adjustment applied the formula:


$$\begin{array}{l} \text{Value in}\;2021\;\mathrm{USD}=\\ \;\;\;\;\;\;\;\text{base year price}\times (\mathrm{CPI}\;\text{in}\;2021/\mathrm{CPI}\;\text{in based year}) \end{array}$$

For this study, CPI in 2021 was 95.27 and CPI in the base year was 95.27 (36).


Direct Medical Costs of Disease(dc)=∑(Ci×Ri)

Our estimates of direct medical costs were presented with and without adjustment for health related imports funded by the Medical Benefits Scheme, namely PET scan and its associated transportation and accommodation costs (37).

### 2.5 Sensitivity analysis

Sensitivity analysis involved use of bounds defined by ±25% of the estimates (31). We did this to make allowance for the possible impact of model uncertainty, enhance understanding to the study’s robustness, as well as accounting for any unrecorded patients by the study sites that are included in the study (31). Moreover, the use of sensitivity analysis enabled us to observe how varying parameter costs affect estimates of direct medical costs (28).

### 2.6 Ethical considerations

Ethical approval for this study was granted by the Antigua and Barbuda Institutional Review Board, Ministry of Health, Wellness, and the Environment (AL-04/052022-ANUIRB), the Institutional Review Board of the Sir Lester Bird Medical Centre, and the University of KwaZulu-Natal Biomedical Research Ethics Committee (BREC/00004531/2022), which waived written informed consent (17). The study adhered to the principles of the Declaration of Helsinki. The principal investigator accessed study data from September 16, 2022, to January 16, 2023 (17), and collected costs data reflecting 2021 price levels between November 22, 2022, to January 25, 2024 (17). In this study, there was no direct patient involvement, and no risk to persons (38). Anonymity was ensured by excluding patient identifiers throughout our data collection process (38).

## 3 Results

### 3.1 Estimation of female breast cancer patients in a single year

Between the years 2017 to 2021, a total of 163 women in Antigua and Barbuda received a confirmed diagnosis of breast cancer. Mean age at presentation was 59 years old (Table 2) (16). Approximately 53% of women were classed in age-group 55–74 years, while 5 and 31% were between ages 20–34 and 35–54 years, respectively (Table 2) (16). Roughly 39% of patients were estimated to have been diagnosed with stage II disease, while 25 and 22% had stages III and IV disease, respectively (Table 2) (39).

**Table 2:** Showing some basic demographic and clinical attributes of the female breast cancer patients, 2017–2021.

Characteristics	Female breast carcinoma (*N* = 163; N (%))
Age at first visit	
Mean patient age (±SD)	58.7 (±13.5)
Mean age 95%CI	56.6–60.8
Median age (IQR)	59.0 (19.0)
Range of Ages	24.0–94.0
Breakdown of age (Years)	
20–34	8 (4.9)
35–54	51 (31.3)
55–74	86 (52.8)
75 and older	18 (11.0)
Clinical stage	
I	24 (14.7)
II	63 (38.7)
III	41 (25.2)
IV	35 (21.5)

According to data from the HID, 22 of the 163 diagnosed female breast cancer patients had died within our study period. By using this value, we estimated that there was an average prevalence of 28 patients of female breast cancer across Antigua and Barbuda during one year of our research period. The identified value was determined by:


$$\text{Patients in single year (cy)}=\frac{\left(\mathrm{Dc}-\mathrm{Na}\right)}{5}$$

*Dc* is number of diagnosed patients = 163

*Na* is the number of patients that died = 22

*cy* is the average annual prevalence cases, defined as patients recorded in a given year = 28

Further, when the tally of patients is increased by 25%, our estimated prevalent patients (cy) = 35

The above approach best provided for an accurate representation of the number of cases requiring cancer care, thus making it crucial to our analysis (31).

### 3.2 Estimated unit costs

**Figure 2:**
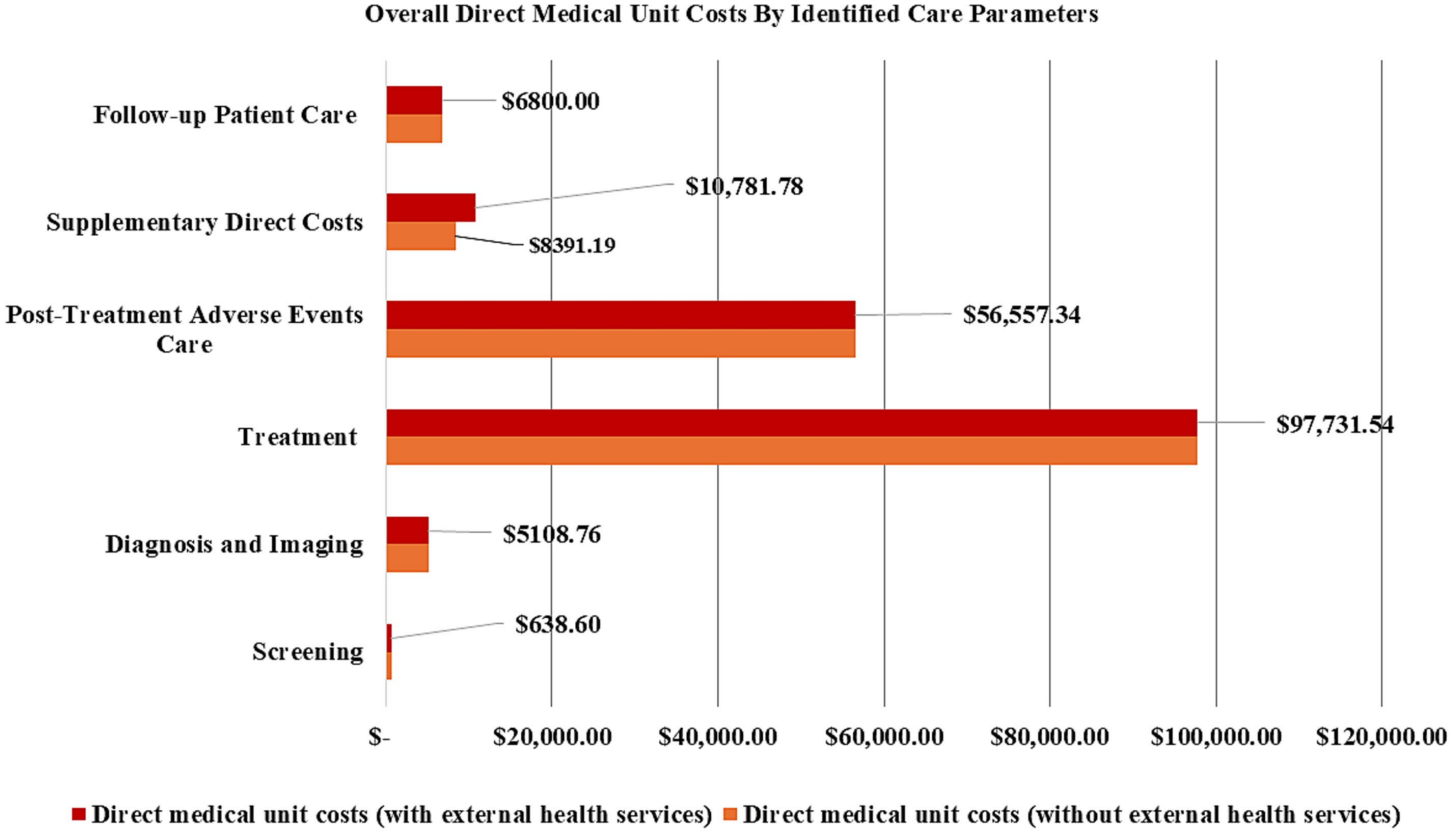
Estimated direct medical unit costs broken down in terms of care parameters.

According to our estimates, the value USD 177618.02 represents for the overall direct-medical unit costs with respect to parameters of screening, imaging and diagnosis, treatment and management of a female breast cancer patient in year 2021 (Table 3). The top three drivers of this costs by care parameter were treatment (USD 97731.54), post-treatment side-effects care (USD 56557.34), and other direct costs (USD 10183.68) (Table 3 and Figure 2). By care components, the major contributors to overall direct medical unit costs included surgery (a combined USD 41486.17), systemic therapy (USD 40013.30), other treatment-related complications (USD 28469.72) and renal issues (USD 20674.86). Estimates of direct medical unit cost related to clinical stage spanned from a low of USD 123673.18 for clinical stage IV disease to highs of USD 165159.35 for clinical stages II and III disease, respectively (Table 3). The direct medical unit cost with respect to clinical stage I disease was USD 157844.25.

**Table 3:** Direct costs for staging, management, and treatment of female breast carcinoma stage I to IV.

Disease stage and treatment attributes	Unit cost (USD)	Unit Cost by Clinical Stages (USD)			
Estimated yearly count of patients (*n* = 28)		Stage I (*n* = 4)	Stage II (*n* = 11)	Stage III (*n* = 7)	Stage IV (*n* = 6)
Screening					
Consultation	$257.65	$257.65	$257.65	$257.65	$257.65
Mammography/Breast ultrasonography	$380.95	$380.95	$380.95	$380.95	$380.95
Diagnosis and imaging					
Biopsy	$2507.27	$2507.27	$2507.27	$2507.27	$2507.27
Imaging (Radiology)	$901.76	$901.76	$901.76	$901.76	$901.76
Laboratory	$629.39	$629.39	$629.39	$629.39	$629.39
Histopathology	$1070.34	$1070.34	$1070.34	$1070.34	$1070.34
Treatment					
Surgery (Lumpectomy)	$15889.32	$15889.32	$15889.32	$15889.32	$-
Surgery (Mastectomy)	$18281.75	$18281.75	$18281.75	$18281.75	$-
Surgery (Axillary Lymph Node Dissection)	$7315.10	$-	$7315.10	$7315.10	$-
External Beam Radiation Therapy	$12974.35	$12974.35	$12974.35	$12974.35	$12974.35
Systemic therapy (Chemotherapy)	$40013.30	$40013.30	$40013.30	$40013.30	$40013.30
Hormonal Therapy	$3257.73	$3257.73	$3257.73	$3257.73	$3257.73
Post-treatment adverse events relieving health services					
Thrombosis prevention	$360.00	$2502.85	$2502.85	$2502.85	$2502.85
Renal Issues	$20674.86	$20674.86	$20674.86	$20674.86	$20674.86
Anemia Management	$6687.76	$6687.76	$6687.76	$6687.76	$6687.76
Infection Control	$365.00	$290.42	$290.42	$290.42	$290.42
Additional treatment-related issues	$28469.72	$13942.77	$13942.77	$13942.77	$13942.77
Supplementary direct costs					
Clinical Nutrition Consultation	$100.00	$100.00	$100.00	$100.00	$100.00
Mental Health Consultation	$128.82	$128.82	$128.82	$128.82	$128.82
Pharmaceutical Services	$89.99	$89.99	$89.99	$89.99	$89.99
Diagnostic Imaging (PET scan performed abroad)†	$991.94	$991.94	$991.94	$991.94	$991.94
Port Placement for Chemotherapy	$7361.33	$7361.33	$7361.33	$7361.33	$7361.33
Medical Response Package (Chemotherapy)	$112.94	$112.94	$112.94	$112.94	$112.94
Travel Support and Housing for Overseas Medical Care (PET Scan) †	$1398.65	$1398.65	$1398.65	$1398.65	$1398.65
Local Transport Services	$561.30	$561.30	$561.30	$561.30	$561.30
Overhead Costs	$36.81	$36.81	$36.81	$36.81	$36.81
Follow-up patient care services					
Post-therapy visits	$1000.00	$1000.00	$1000.00	$1000.00	$1000.00
Computed Tomography with Contrast Agent	$1900.00	$1900.00	$1900.00	$1900.00	$1900.00
Blood Count with Differential Analysis	$1460.00	$1460.00	$1460.00	$1460.00	$1460.00
Basic Metabolic Panel (7 tests)	$180.00	$180.00	$180.00	$180.00	$180.00
Kidney Profile (Panel 1)	$780.00	$780.00	$780.00	$780.00	$780.00
Hepatic Panel	$680.00	$680.00	$680.00	$680.00	$680.00
Haemoglobin A1c	$90.00	$90.00	$90.00	$90.00	$90.00
Total Serum Cholesterol	$160.00	$160.00	$160.00	$160.00	$160.00
Echocardiogram	$300.00	$300.00	$300.00	$300.00	$300.00
Chest X-ray	$250.00	$250.00	$250.00	$250.00	$250.00
Total (without external health services)	$175227.44	$155453.66	$162768.76	$162768.76	$121282.59
Total (with external health services)	$177618.03	$157159.35	$165159.35	$165159.35	$123673.18

†External health services (cancer care services along with overseas related healthcare).

Generally, the overall estimates of direct medical unit costs, inclusive of costs per disease stage, showed a nominal reduction by USD 2390.59 when the costs of external health services (PET scan and its associated transportation/accommodation costs) were excluded from our calculations (Table 3).

### 3.3 Estimated annual direct medical costs

**Figure 3:**
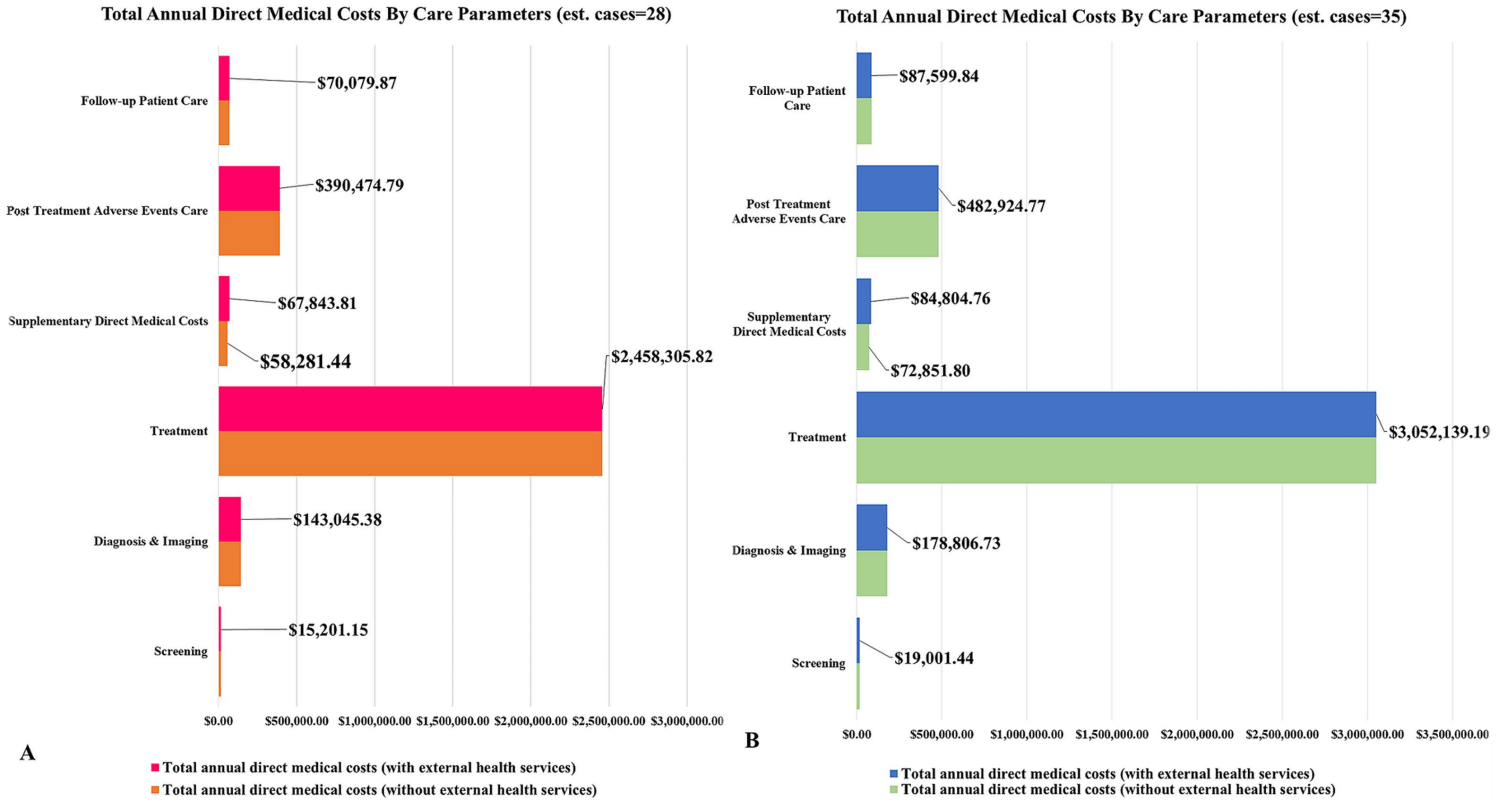
**(A,B)** The contributors to estimated annual direct medical costs disaggregated by care parameters estimated patients in a single year.

Estimated annual direct medical costs of female breast cancer based on our assessment was USD 3144950.82 (ranging between USD 2358713.11 − USD 3931188.52). With the non inclusion of external health services, this valued was estimated at USD 3135388.45 (range USD 2 351,541.34 − USD 3919235.56) (Table 4). The chief contributors to our estimate of total annual direct medical costs (with external health services) were treatment (USD 2458305.82), post-treatment care (USD 390474.79), and diagnosis and imaging (USD 143045.38) (Table 4 and Figure 3A).

**Table 4:** Estimated total annual costs of female breast carcinoma -direct medical costs.

Identified parameters	Care components	Estimated yearly count of patients (*n* = 28)	Estimate of average cost in 2021 (USD)	Total annual costs (USD)	Aggregate costs and relative percentage (adjusted)	Range of total annual costs (USD) ± 25%	
						Lower limit	Upper limit
Screening	Screening						
	Consultation (Clinical assessment/Physical examination)	28	$257.65	$7214.10		$5410.58	$9017.63
	Mammogram/Breast Ultrasonography	28	$138.02	$3864.70		$2898.52	$4830.87
	Breast Ultrasound	28	$147.23	$4122.35		$3091.76	$5152.93
Subtotal				$15201.15	0.48%	$11400.86	$19001.44
Diagnosis and imaging	Diagnosis						
	Biopsy	28	$2507.27	$70203.54		$52652.66	$87754.43
	Complete Blood Panel	28	$629.39	$17623.03		$13217.27	$22028.78
	Histopathology	28	$1070.34	$29969.45		$22477.09	$37461.81
	Imaging Studies	28	$901.76	$25249.37		$18937.02	$31561.71
Subtotal				$143045.38	4.55%	$107284.04	$178806.73
Treatment	Treatment						
	Stage I	4	$90416.44	$361665.77		$271249.33	$452082.21
	Stage II	11	$97731.54	$1075046.98		$806285.23	$1343808.72
	Stage III	7	$97731.54	$684120.81		$513090.60	$855151.01
	Stage IV	6	$56245.38	$337472.26		$253104.20	$421840.33
Subtotal				$2458305.82	78.17%	$1843729.36	$3072882.27
Post-treatment adverse events relieving health services	Post-treatment adverse events relieving health services						
	Thrombosis prevention	28	$132.50	$3710.11		$2782.58	$4637.64
	Renal Issues	1	$20674.86	$20674.86		$15506.14	$25843.57
	Anaemia Management	28	$2461.54	$68923.14		$51692.36	$86153.93
	Infection Control	28	$134.34	$3761.64		$2821.23	$4702.05
	Additional Treatment-related Issues	28	$10478.75	$293405.04		$220053.78	$366756.30
Subtotal				$390474.79	12.42%	$292856.09	$488093.49
Supplementary direct medical costs	Supplementary direct costs						
	Clinical Nutrition Consultation	28	$100.00	$2800.00		$2100.00	$3500.00
	Mental Health **C**onsultation	28	$128.82	$3607.05		$2705.29	$4508.82
	Pharmaceutical Services	28	$89.99	$2519.78		$1889.84	$3149.73
	Diagnostic Imaging (PET scan performed abroad)†	4	$991.94	$3967.76		$2975.82	$4959.70
	Port Placement for Chemotherapy	4	$7361.33	$29445.32		$22083.99	$36806.65
	Medical Response Package (Chemotherapy)	28	$112.94	$3162.25		$2371.69	$3952.81
	Travel Support and Housing for Overseas Medical Care (PET Scan) †	4	$1398.65	$5594.61		$4195.96	$6993.26
	Local Transport Services	28	$561.30	$15716.44		$11787.33	$19645.55
	Overhead Costs	28	$36.81	$1030.59		$772.94	$1288.23
Subtotal				$67843.81	2.16%	$50882.86	$84804.76
Follow-up patient care services	Follow-up patient care services						
	**Post-**therapy visits	28	$368.07	$10305.86		$7729.40	$12882.33
	Computed Tomography with contrast agent	28	$699.33	$19581.14		$14685.86	$24476.43
	Blood Count with Differential Analysis	28	$537.38	$15046.56		$11284.92	$18808.20
	Basic Metabolic Panel (7 tests)	28	$66.25	$1855.06		$1391.29	$2318.82
	Kidney Profile (Panel 1)	28	$287.09	$8038.57		$6028.93	$10048.22
	Hepatic Panel	28	$250.29	$7007.99		$5255.99	$8759.98
	Haemoglobin A1c	28	$33.13	$927.53		$695.65	$1159.41
	Total Serum Cholesterol	28	$58.89	$1648.94		$1236.70	$2061.17
	Echocardiogram	28	$110.42	$3091.76		$2318.82	$3864.70
	Chest X-ray	28	$92.02	$2576.47		$1932.35	$3220.58
Subtotal				$70079.87	2.23%	$52559.90	$87599.84
Total direct medical costs (without external health services)				$3135388.45		$2351541.34	$3919235.56
Total direct medical costs (with external health services)				$3144950.82		$2358713.11	$3931188.52
Total direct medical costs (with external health services)-A				$1915797.91		$1436848.43	$2394747.39
Total direct medical costs (with external health services)-B				$4717426.22		$3538069.67	$5896782.78
Total direct medical costs (with external health services)-C				$3905276.72		$2928957.54	$4881595.90

†External health services (overseas offered Pet scan and associated travelling and accommodation services). A: with adjustments to total direct medical costs indicating a 50% decline in direct medical unit cost of treatment per each stage of cancer. B: with adjustments to total direct medical costs showing a 50% rise in direct medical unit costs for all components of cancer care. C: with adjustments to total direct medical costs representing a 25% rise in the estimated number of patients in a single year (from 28 to 35).

Where external health services were not included in the assessment, other- direct costs were reduced to USD 58281.44 (range USD 43711.08 − USD 72851.80), and overall annual direct medical costs cut by USD 9,562.37 (range USD 7171.78 − USD 11952.96).

Further, by decreasing direct medical unit cost per clinical stage by 50% so as to assess its effect on treatment costs and the annual estimates, showed that the overall annual direct medical costs declined to USD 1915797.91 (spanning between USD 1436848.43 − USD 2394747.39).

When the direct medical unit costs of the care components was increased by 50%, our estimate of total annual direct costs became USD 4717426.22 (ranging between USD 3538069.67 − USD 5896782.78), which represented a 50% increase above that of our initial annual estimates.

Increasing our average number of prevalence cases by 25% (i.e., 28 to 35), resulted in a 24% increase in our estimated total annual direct medical cost. The resulting value was USD 3905276.72 (ranging between USD 2928957.54 − USD 4881595.90) (Table 4 and Figure 3B). This implied that every extra prevalent case beyond the established number of 28 could likely add around USD 108617.99 to our estimated annual direct cost. Following the removal of external health services from this total, the estimated value dropped to USD 3893323.76 (ranging between USD 2919992.82 − USD 4866654.70), while also showing no appreciable change in the percentage ranking of our cost parameters.

## 4 Discussion

Our investigation quantified the economic impact of female breast cancer in Antigua and Barbuda by estimating the total annual direct medical costs from the standpoint of the healthcare provider (29). The yearly direct medical costs of this cancer in women in the country was approximately USD 3144950.82 (ranging between USD 2358713.11 − USD 3931188.52) in 2021, with the leading contributors to these costs being treatment (USD 2458305.82), post-treatment side-effects care (USD 390474.79), and diagnosis and imaging care (USD 143045.38) in that order. This amount represents about 0.2% of the country’s 2021 GDP (current USD) of USD 1.6 billion (40). Even when the external health services of overseas PET scan was removed from the analysis, our estimates position relative to GDP (current USD) remained unchanged at USD3.1 million, thus affirming the inclusion of external health services in the cost estimates (37).

Considering the population size and estimate of prevalent cases (28 patients in a single year) our values underscore the substantial burden, both epidemiologically and economically, that treating and managing female breast cancer places on both the health sector and the resources of health in general in Antigua and Barbuda. This is so especially when viewed alongside the country’s per capita health expenditure and budgetary allocations (40, 41).

This study tells us that, all things being equal, should the island’s prevalent cases increase from 28 to 35 then we can reasonably expect a major shift in the yearly direct medical costs from an initial USD 3.1 million to approximately USD 3.9 million (ranging between USD 2.9 million−USD 4.9 million). This suggests a modest USD 108617.99 increase in total annual direct costs for each added patient to our model, and thus, further emphasizing the economic impact of our studied female cancer to the country’s health system.

Treatment was the chief cost driver, accounting for 78% (USD 2458305.82) of total annual costs, with costs associated with stages II and III disease being notably higher than those of stages I and IV disease, respectively. Considering the influence of the guidelines for female breast cancer care in Antigua and Barbuda (23, 24), these findings could be the due to patients with stage II and III disease experiencing more intensive and frequent treatments such as surgery, radiation, and chemotherapy, when compared to stage IV patients, who may receive more palliative care (42, 43). These findings are suggestive of the need to reorient health policy towards prevention and early interventions in disease management. Further, though this observation is consonant with variableness in costs, and is dependent on the methodology employed, it appears inconsistent with the observation of a few studies which showed that treatment costs become incrementally higher with an increasing disease stage at diagnosis (44–46). For example, Teich et al., in investigating the cost impact of managing breast cancer within Brazilian clinical practice, and Davari et al., in looking at direct medical costs of services received in Iran, both observed that direct costs per patient increased according to clinical stage (44, 45), while Blumen and colleagues in comparing costs by tumor stage and service type, suggested that early-stage diagnosis is associated with reduced costs to the healthcare system compared to late-stage diagnosis (46). Additionally, our findings affirmed treatment as the primary cost driver across all four disease stages, an observation that was found congruent with that of several studies, inclusive of a study by Mousa et al., which assessed the full direct medical costs of breast cancer in Jordan from the provider’s standpoint (47).

Other leading drivers of cost, post-treatment side-effects care, which accounted for 12% (US$390474.79) of total annual costs, reflects the costs associated with the toll of varying consequences, such as renal issues and ‘other treatment complications’, on patients, following breast cancer treatment (48). Even though our estimates for diagnosis and imaging represented only 5% of the total annual cost, it indicates costs of the higher consumption of resources required for these procedures (49). This underscores the need to adopt cost containment strategies to help control future costs associated with detection and management of breast cancer occurring in the women of Antigua and Barbuda.

Leading drivers under direct medical unit costs were systemic therapy, surgery and radiotherapy, and complications of treatment, and for diagnosis and imaging, biopsy and histopathology costs. These costs are reflective of the likely differences in the costs components within each parameter and is indicative of inherent variations in managing females diagnosed with this specific cancer within the local setting (50).

Overall, a further examination of treatment costs accentuated its position as a major cost driver of direct medical costs of cancer of the breast diagnosed in women of Antigua and Barbuda. This position was similarly highlighted in a study (i) by Atieno et al., which highlighted that cost related to treating cancer of the breast in Kenya is significantly higher when compared to the cost of treating other common cancers (51), and (ii) by Dahlberg et al., in Sweden where treatment costs (costs including medical treatment, surgery, and radiotherapy combined) were shown to comprise the largest component of total costs of breast cancer care (52).

There were several strengths associated with this study. Firstly, this study presents a detailed assessment of cost data and corresponding care components that contribute to the economic burden of diagnosed cancer of the breast in women of Antigua and Barbuda. When combined with inputs from healthcare experts, namely, the medical oncologist, pathologist and oncology nurses, our values reasonably reflect the direct cost of screening, diagnosing and imaging, care and management of female breast carcinoma from the standpoint of the healthcare provider in the country. This, therefore, confirms our confidence in the estimates as being a reliable representation of the direct medical costs of breast cancer among women in the country. Secondly, our study highlighted the nonexistence of an established registry for cancer cases, a database on healthcare expenditures, a national breast cancer plan, nationally organized screening initiatives for early diagnosis, and legal framework that ensures mandatory reporting of cancer patients across all levels of care on the island (2, 17, 27, 49). Besides facilitating the assessment of the epidemiological burden of female breast carcinoma, functional cancer registries, similar to those in the Caribbean countries of Martinique and Puerto Rico, would help in documenting prevalence cases and describing local distribution of female breast carcinoma patients, thereby providing organized data that could be used in future economic evaluation studies on female breast and other common cancers in the country. Further, in view of the usefulness regarding the prevalence-based methodology for our study, our results highlight its importance as a costing approach that can be easily replicated not just for cancers but also for other disease conditions in Antigua and Barbuda and in similarly low-resourced states in the Eastern Caribbean and wider Caribbean regions.

A limitation of this study is that it was done from the healthcare provider’s perspective and considered just direct medical costs. As such it never evaluated the role of other forms of costs, such as indirect costs, loss productivity, quality of life, and end-of-life care and out-of-pocket expenses (49). Future studies could examine the effect of these and other cost dimensions pertaining to the economic burden of breast carcinoma in Antigua and Barbuda (49, 50). Additionally, our approach to estimating annual prevalent cases may not have accurately accounted for variations in survivorship durations or possible under-ascertainment of cases (28). This could have had an untold effect on our prevalence estimates and subsequent cost analysis (28). To address this, the study employed one-way sensitivity analysis (17). In the absence of national treatment guidelines, the study considered experts’ input under local patient care treatment protocols, and this may have potentially introduced bias in our estimates because of variableness in management practices. Despite the noted constraints, we are assured that our research presents an accurate reflection of the prevailing cost of illness associated with breast cancer among women in Antigua and Barbuda.

Taking into account the expected increase in the occurrence of female breast cancer in years to come for states such as Antigua and Barbuda, managing breast cancer among women could become an overwhelming undertaking for the healthcare provider (16). The results of our research imply the necessity to adopt several cost containment strategies, to include sourcing lower cost drug therapies that could help reduce treatment costs related to breast cancer care (49). Additionally, our study results could be used as baseline information to appraise the cost-effectiveness related to programmatic activities linked to identification, treatment, and the management of female breast cancer as well as to develop evidence-based guidelines aimed at providing affordable cancer treatment at all levels of healthcare practice in the country (13, 50).

## 5 Conclusion

Carcinoma of the breast is among the life-altering disorders that affects the population of women within Antigua and Barbuda. Our research presents the first of any evaluation of the economic burden of female breast carcinoma in the nation. Costs estimates derived are considerable in view of the local realities. Among primary contributors to costs are treatment, and post-treatment adverse events relieving health services. Aside from re-orienting care to early detection and prevention as measures to aid cost containment and to manage the financial burden of female breast cancer, obtaining data from a population-based registry, and conducting further economic assessments through cost-effectiveness studies and other forms of cost of illness assessments such as from the individual, and societal perspectives would be useful. Our study results, could also serve as a starting point for developing health policies, crafting resources utilization guidelines and initiating certain budget-related decisions surrounding breast cancer care in Antigua and Barbuda.

## Data Availability

The original contributions presented in the study are included in the article/supplementary material, further inquiries can be directed to the corresponding author/s.

## References

[1] Bray F Laversanne M Sung H Ferlay J Siegel RL Soerjomataram I . Global cancer statistics 2022: GLOBOCAN estimates of incidence and mortality worldwide for 36 cancers in 185 countries. CA Cancer J Clin. (2024) 74:229–63. doi: 10.3322/caac.21834, 38572751

[2] Simon L Gaskin P Daniel G Samuel J Goodwin S. Antigua/Barbuda cancer incidence study. WIMJ Open. (2014) 1:84–7. doi: 10.7727/wimjopen.2014.072

[3] Luo Q Smith DP. Global cancer burden: progress, projections, and challenges. Lancet. (2025) 406:1536–7. doi: 10.1016/S0140-6736(25)01570-3, 41015053

[4] Franklin M Pollard D Sah J Rayner A Sun Y Dube F . Direct and indirect costs of breast Cancer and associated implications: A systematic review. Adv Ther. (2024) 41:2700–22. doi: 10.1007/s12325-024-02893-y, 38833143 PMC11213812

[5] Chen S Cao Z Prettner K Kuhn M Yang J Jiao L . Estimates and projections of the global economic cost of 29 cancers in 204 countries and territories from 2020 to 2050. JAMA Oncol. (2023) 9:465–72. doi: 10.1001/jamaoncol.2022.7826, 36821107 PMC9951101

[6] Mariotto AB Robin Yabroff K Shao Y Feuer EJ Brown ML. Projections of the cost of cancer care in the United States: 2010-2020. JNCI J Natl Cancer Inst. (2011) 103:117–28. doi: 10.1093/jnci/djq495, 21228314 PMC3107566

[7] Kim YA Oh I-H Yoon S-J Kim H-J Seo H-Y Kim E-J . The economic burden of breast Cancer in Korea from 2007-2010. Cancer Res Treat. (2015) 47:583–90. doi: 10.4143/crt.2014.143, 25687860 PMC4614197

[8] Seweryn M Banas T Augustynska J Lorenc O Kopel J Pluta E . The direct and indirect costs of breast cancer in Poland: estimates for 2017–2019. Int J Environ Res Public Health. (2022) 19:16384. doi: 10.3390/ijerph192416384, 36554267 PMC9778099

[9] Palacios A Rojas-Roque C González L Bardach A Ciapponi A Peckaitis C . Direct medical costs, productivity loss costs and out-of-pocket expenditures in women with breast cancer in Latin America and the Caribbean: a systematic review. PharmacoEconomics. (2021) 39:485–502. doi: 10.1007/s40273-021-01014-9 33782865

[10] The World Bank Group. Economic Review | Latin America and the Caribbean October 2025. World Bank IBRD-IDA (2025). Available online at: https://www.worldbank.org/en/region/lac/publication/perspectivas-economicas-america-latina-caribe (Accessed December 1, 2025)

[11] Matsumoto K Haga K Kitazawa T Seto K Fujita S Hasegawa T. Cost of illness of breast cancer in Japan: trends and future projections. BMC Res Notes. (2015) 8:539. doi: 10.1186/s13104-015-1516-y, 26438238 PMC4593212

[12] Jain M Mukherjee K. Economic burden of breast cancer to the households in Punjab, India. Int J Med Public Health. (2016) 6:13. doi: 10.4103/2230-8598.179754

[13] Afkar A Jalilian H Pourreza A Mir H Sigaroudi AE Heydari S. Cost analysis of breast cancer: a comparison between private and public hospitals in Iran. BMC Health Serv Res. (2021) 21:219. doi: 10.1186/s12913-021-06136-6, 33706762 PMC7953682

[14] Okyere Asante PG Owusu AY Oppong JR Amegah KE Nketiah-Amponsah E. An assessment of the direct and indirect costs of breast cancer treatment in leading cancer hospitals in Ghana. PLoS One. (2024) 19:e0301378. doi: 10.1371/journal.pone.0301378, 38771827 PMC11108162

[15] Caribbean Development Bank. Caribbean Economic Review and Outlook 2024–2025. Caribb Dev Bank (2025). Avaialble online at: https://www.caribank.org/publications-and-resources/resource-library/economic-reviews/caribbean-economic-review-and-outlook-2024-2025 (Accessed December 1, 2025).

[16] Bovell AAN Ramaliba T Goodwin SO Phillip JC Ncayiyana J Ginindza TG. Incidence, trends and patterns of female breast, cervical, colorectal and prostate cancers in Antigua and Barbuda, 2017–2021: a retrospective study. BMC Cancer. (2025) 25:72. doi: 10.1186/s12885-025-13459-8, 39806280 PMC11727155

[17] Bovell AAN Ngcamphalala C Rhudd A Ncayiyana J Ginindza TG. The economic burden of prostate Cancer in Antigua and Barbuda: A prevalence-based cost-of-illness analysis from the healthcare provider perspective. Int J Environ Res Public Health. (2024) 21:1527. doi: 10.3390/ijerph21111527, 39595794 PMC11593963

[18] Bovell AAN Ngcamphalala C Abbott D Ncayiyana J Ginindza TG. Cost analysis related to diagnosis, treatment and Management of Cervical Cancer in Antigua and Barbuda: A prevalence-based cost-of-illness study. Int J Environ Res Public Health. (2024) 21:1685. doi: 10.3390/ijerph21121685, 39767524 PMC11675278

[19] Commonwealth Network. Find Health and Medical Expertise in Antigua and Barbuda. Heal Med Antig Barbuda (2020). Avaialble online at: https://www.commonwealthofnations.org/sectors-antigua_and_barbuda/business/health_and_medical/ (Accessed September 22, 2023)

[20] Communications and Marketing Department Medical Benefits Scheme. About Us. Med Benefits Scheme (2023). Avaialble online at: https://www.mbs.gov.ag/v2/about/ (Accessed September 22, 2023)

[21] World Health Organization. International Statistical Classification of Diseases and Related Health Problems, 10th Revision. 5th ed. Geneva: World Health Organization: WHO Library Cataloguing-in-Publication Data (2016).

[22] Stollenwerk B Welchowski T Vogl M Stock S. Cost-of-illness studies based on massive data: a prevalence-based, top-down regression approach. Eur J Health Econ. (2016) 17:235–44. doi: 10.1007/s10198-015-0667-z, 25648977

[23] World Health Assembly 70. Cancer Prevention and Control in the Context of an Integrated Approach, Geneva PP. Geneva: World Health Organization (2017).

[24] Gradishar WJ Anderson BO Abraham J Aft R Agnese D Allison KH . Breast Cancer, version 3.2020, NCCN clinical practice guidelines in oncology. J Natl Compr Cancer Netw. (2020) 18:452–78. doi: 10.6004/jnccn.2020.0016, 32259783

[25] Cavalli F Kaye SB Hansen HH Armitage JO. "Breast Cancer". In: Cavalli F SB K` Hansen HH Armitage JO Piccart-Gebhart M, editors. Textbook of Medical Oncology, 4th Edn. Baton Rouge: Taylor & Francis (2009). p. 77–105.

[26] Hao S Östensson E Eklund M Grönberg H Nordström T Heintz E . The economic burden of prostate cancer – a Swedish prevalence-based register study. BMC Health Serv Res. (2020) 20:448. doi: 10.1186/s12913-020-05265-8 32434566 PMC7238534

[27] Rhudd AR. The current state of prostate cancer in Antigua & Barbuda-2021. Ecancermedicalscience. (2021) 15:ed112. doi: 10.3332/ecancer.2021.ed112, 34567266 PMC8426014

[28] Jo C. Cost-of-illness studies: concepts, scopes, and methods. Clin Mol Hepatol. (2014) 20:327–37. doi: 10.3350/cmh.2014.20.4.327, 25548737 PMC4278062

[29] Clabaugh G Ward MM. Cost-of-illness studies in the United States: a systematic review of methodologies used for direct cost. Value Health. (2008) 11:13–21. doi: 10.1111/j.1524-4733.2007.00210.x, 18237356

[30] Larg A Moss JR. Cost-of-illness studies: a guide to critical evaluation. PharmacoEconomics. (2011) 29:653–71. doi: 10.2165/11588380-000000000-00000 21604822

[31] Ginindza TG Sartorius B Dlamini X Östensson E. Cost analysis of human papillomavirus-related cervical diseases and genital warts in Swaziland. PLoS One. (2017) 12:e0177762. doi: 10.1371/journal.pone.0177762, 28531205 PMC5439687

[32] Drummond M Sculpher MJ Claxton K Stoddart GL Torrance GW HLSTA-TT. Methods for the economic evaluation of health care programmes (2015). Avaialble online at: https://ukzn.on.worldcat.org/oclc/92981392 (Accessed January 29, 2024).

[33] Hodgson Thomas A Meiners Mark R. Cost-of-illness methodology: a guide to current practices and procedures. Milbank Mem Fund Q. (1982) 60:429. doi: 10.2307/3349801 6923138

[34] Chapel JM Wang G. Understanding cost data collection tools to improve economic evaluations of health interventions. Stroke Vasc Neurol. (2019) 4:214–22. doi: 10.1136/svn-2019-000301, 32030205 PMC6979867

[35] Amin MB Greene FL Edge SB Compton CC Gershenwald JE Brookland RK . The eighth edition AJCC cancer staging manual: continuing to build a bridge from a population-based to a more “personalized” approach to cancer staging. CA Cancer J Clin. (2017) 67:93–9. doi: 10.3322/caac.21388, 28094848

[36] Statistic Division Ministry of Finance the Economy Public Administration Public Broadcasting and Information. Consumer Price Index. Stat Div Minist Financ Antig Barbuda (2022). Avaialble online at: https://statistics.gov.ag/wp-content/uploads/2022/01/Monthly-CPI-December-2021.pdf (Accessed March 8, 2024)

[37] OECD/Eurostat/WHO. A System of Health Accounts 2011: Revised ed. Paris: OECD Publishing (2017).

[38] Ekdahl Hjelm T Matovu A Mugisha N Löfgren J. Breast cancer care in Uganda: A multicenter study on the frequency of breast cancer surgery in relation to the incidence of breast cancer. PLoS One. (2019) 14:e0219601. doi: 10.1371/journal.pone.0219601, 31295322 PMC6622523

[39] Hennis AJ Hambleton IR Wu S-Y Leske MC Nemesure B. Breast cancer incidence and mortality in a Caribbean population: comparisons with African-Americans. Int J Cancer. (2009) 124:429–33. doi: 10.1002/ijc.23889, 18844211 PMC2659611

[40] The World Bank. Antigua and Barbuda. World Bank IBRD-IDA (2024). Avaialble online at: https://data.worldbank.org/country/AG (Accessed April 15, 2024)

[41] Browne G. Antigua and Barbuda 2021 Budget Statement “Maintaining a Healthy Nation and Restoring a Vibrant Economy.” Saint John’s, Antigua and Barbuda: Government of Antigua and Barbuda (2021). Available at: https://ab.gov.ag/pdf/budget/Budget_Speech_2021.pdf (Accessed September 22, 2023).

[42] Gąska I Czerw A Pajewska M Partyka O Deptała A Badowska-Kozakiewicz A . The cost of breast Cancer: economic and social perspective. Cancers (Basel). (2025) 17:3012. doi: 10.3390/cancers17183012, 41008856 PMC12468891

[43] Sun L Legood R Dos-Santos-Silva I Gaiha SM Sadique Z. Global treatment costs of breast cancer by stage: a systematic review. PLoS One. (2018) 13:e0207993. doi: 10.1371/journal.pone.0207993, 30475890 PMC6258130

[44] Teich N Pepe C Vieira FM Teich V Cintra M Leibel F . Retrospective cost analysis of breast cancer patients treated in a Brazilian outpatient cancer center (OCC). J Clin Oncol. (2010) 28:e11026. doi: 10.1200/jco.2010.28.15_suppl.e11026

[45] Davari M Yazdanpanah F Aslani A Hosseini M Nazari AR Mokarian F. The direct medical costs of breast cancer in Iran: analyzing the patient’s level data from a cancer specific hospital in Isfahan. Int J Prev Med. (2013) 4:748–54. doi: 10.4103/2008-7802.117079 24049592 PMC3775213

[46] Blumen H Fitch K Polkus V. Comparison of treatment costs for breast cancer, by tumor stage and type of service. Am Health Drug Benefits. (2016) 9:23–32. 27066193 PMC4822976

[47] Mousa R Hammad E Melhem J Al-Jaghbir M. Direct medical costs of breast cancer in Jordan: cost drivers and predictors. Expert Rev Pharmacoecon Outcomes Res. (2021) 21:647–54. doi: 10.1080/14737167.2021.1859372, 33353434

[48] Tommasi C Balsano R Corianò M Pellegrino B Saba G Bardanzellu F . Long-term effects of breast Cancer therapy and care: calm after the storm? J Clin Med. (2022) 11:11. doi: 10.3390/jcm11237239, 36498813 PMC9738151

[49] Alghamdi A Balkhi B Alqahtani S Almotairi H. The economic burden associated with the management of different stages of breast cancer: a retrospective cost of illness analysis in Saudi Arabia. Healthcare. (2021) 9:907. doi: 10.3390/healthcare9070907, 34356285 PMC8307453

[50] Luengo-Fernandez R Leal J Gray A Sullivan R. Economic burden of cancer across the European Union: a population-based cost analysis. Lancet Oncol. (2013) 14:1165–74. doi: 10.1016/S1470-2045(13)70442-X, 24131614

[51] Atieno OM Opanga S Martin A Kurdi A Godman B. Pilot study assessing the direct medical cost of treating patients with cancer in Kenya; findings and implications for the future. J Med Econ. (2018) 21:878–87. doi: 10.1080/13696998.2018.1484372, 29860920

[52] Dahlberg L Lundkvist J Lindman H. Health care costs for treatment of disseminated breast cancer. Eur J Cancer. (2009) 45:1987–91. doi: 10.1016/j.ejca.2009.03.023, 19398326

